# Genetic Variants of *LRRK2* in Taiwanese Parkinson’s Disease

**DOI:** 10.1371/journal.pone.0082001

**Published:** 2013-12-05

**Authors:** Yih-Ru Wu, Kuo-Hsuan Chang, Wen-Teng Chang, Ya-Chin Hsiao, Hsuan-Chu Hsu, Pei-Ru Jiang, Yi-Chun Chen, Chih-Ying Chao, Yi-Chung Chang, Bo-Hsun Lee, Fen-Ju Hu, Wan-Ling Chen, Guey-Jen Lee-Chen, Chiung-Mei Chen

**Affiliations:** 1 Department of Neurology, Chang Gung Memorial Hospital, Chang Gung University College of Medicine, Taipei, Taiwan; 2 Department of Life Science, National Taiwan Normal University, Taipei, Taiwan; Oslo University Hospital, Norway

## Abstract

Genetic variants of leucine-rich repeat kinase 2 (*LRRK2*) were reported to alter the risk for Parkinson’s disease (PD). However, the genetic spectrum of *LRRK2* variants has not been clearly disclosed yet in Taiwanese population. Herein, we sequenced *LRRK2* coding region in 70 Taiwanese early onset PD patients (age at onset ≤ 50), and found six amino acid-changing single nucleotide polymorphisms (SNPs, N551K, R1398H, R1628P, S1647T, G2385R and M2397T), one reported (R1441H) and 2 novel missense (R767H and S885N) mutations. We examined the frequency of identified *LRRK2* variants by genotyping 573 Taiwanese patients with PD and 503 age-matched control subjects. The results showed that PD patients demonstrated a higher frequency of G2385R A allele (4.6%) than control subjects (2.1%; odds ratio = 2.27, 95% confidence interval: 1.38–3.88, *P* = 0.0017). Fewer PD patients (27.7%) carried the 1647T-2397T haplotype as compared with the control subjects (33.0%; odds ratio = 0.80, 95% confidence interval: 0.65–0.97, *P* = 0.0215). However, the frequency of 1647T-2385R-2397T haplotype (4.3%) in PD patients was still higher than in control subjects (1.9%, odds ratio: 2.15, 95% confidence interval: 1.27–3.78, *P* = 0.0058). While no additional subject was found to carry R767H and R1441H, one more patient was observed to carry the S885N variant. Our results indicate a robust risk association regarding G2385R and a new possible protective haplotype (1647T-2397T). Gene-environmental interaction and a larger cohort study are warranted to validate our findings. Additionally, two new missense mutations (R767H and S885N) regarding *LRRK2* in PD patients were identified. Functional studies are needed to elucidate the effects of these *LRRK2* variants on protein function.

## Introduction

Parkinson’s disease (PD) is the second most common neurodegenerative disorder in the world [1]. It affects 1% of the population aged over sixty, and is characterized by a slowness of movement (bradykinesia) and a difficulty in initiating movement (akinesia) [1]. The pathogenesis of PD is associated with progressive degeneration of dopaminergic (DA) neurons and the presence of eosinophilic cytoplasmic inclusion bodies (Lewy bodies) with enrichment of α-synuclein in the ventral midbrain [2]. 

The etiology of PD remains to be explored. Mutations in the gene for leucine-rich repeat kinase 2 (*LRRK2*) account for some patients with autosomal dominantly inherited PD [3,4]. *LRRK2* gene encodes a large multidomain protein that includes ANK (ankyrin repeat), LRR (leucine-rich repeat), ROC (Ras of complex proteins; GTPase), COR (C-terminal of ROC), MAPKKK (mitogen-activated kinase kinase kinase) and WD40 domains [5,6]. Up to now, a number of putatively mutations and single nucleotide polymorphisms (SNPs) in the *LRRK2* gene have been reported (the Human Gene Mutation Database, http://www.hgmd.cf.ac.uk/ac/index.php?gene=LRRK2).

In Taiwan, the *LRRK2* G2385R and R1628P variants may play significant roles in susceptibility to PD [7–10]. In contrast, *LRRK2* G2019S, a common mutation amongst PD patients in North America, Europe and North Africa [3,4,11–14], has not been found in Taiwanese PD patients [15]. The disease penetrance for G2019S carriers is age dependent, increasing from less than 20% at age 50 years or younger to 80~85% at age 70 years [16,17]. Age at onset (AAO) of mutation carriers is broad, ranged from 28 to 73 years, and mutation carriers were clinically indistinguishable from idiopathic PD [18,19]. To further examine the genetic variations of *LRRK2* in Taiwanese PD, we sequenced the *LRRK2* coding region in 70 Taiwanese PD patients and assessed the association of identified SNPs with the risk of PD by utilizing a large case-control cohort of patients and controls, to provide more insight into *LRRK2* variants in Taiwanese PD patients.

## Results

### Mutation analysis of LRRK2

**Figure 1:**
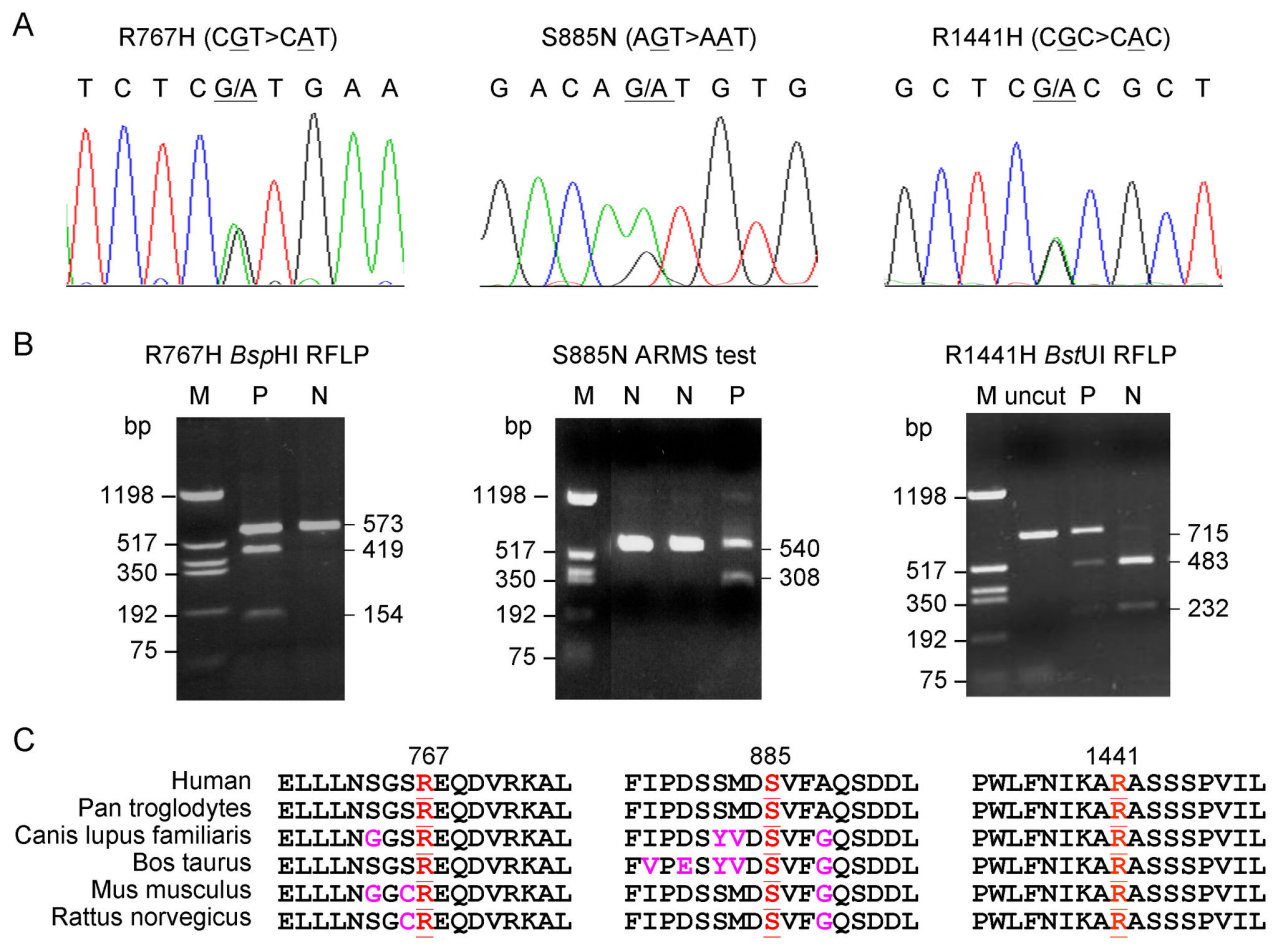
Mutation identification and amino acid sequence alignment. **a** Chromatograms of direct cDNA sequencing of R767H, S885N and R1441H. **b** Restriction enzyme RFLP or ARMS analysis of R767H, S885N, R1441H mutations. On agarose gel, R767H results restriction by *Bsp*HI and leads to additional 419 and 154 bp bands, whereas R1441H prevents restriction by *Bst*UI and leads to an additional 715 bp band. **c** Evolutionary conservation of the regions of LRRK2 R767H, S885N and R1441H using the program Vector NTI.

*LRRK2* cDNA fragments encompassing ANK to WD40 domains from 70 patients with the age at onset of PD ≤ 50 were amplified for sequence analysis. In addition to twelve exonic variants (N551K, L953, R1398H, K1423, G1624, R1628P, K1637, S1647T, G1819, E2108, G2385R and M2397T) (Table 1), one reported (R1441H) [20–23] and five novel (R767H and S885N in Figure 1A; R1483, Y2018 and N2047, data not shown) variants were identified. The three missense substitutions were then examined using PCR-based *Bsp*HI RFLP (R767H), ARMS test (S885N), or *Bst*UI RFLP (R1441H) (Figure 1B) in PD patients (n = 612) and controls (n = 508). While no additional subject was found to carry R767H and R1441H, one more patient was observed as carrying the S885N variant. No controls were observed carrying the novel variants R767H and S885N. R767H, S885N, and R1441H are located in the ANK, in between ANK and LRR, and in the ROC domain, respectively. The three missense variants are highly conserved among the known mammalian homologues of the LRRK2 protein (Figure 1C).

**Table 1:** Exonic variants identified in early-onset PD.

**Exon**	**Accession no.**	**Amino acid (nucleotide) change**	**Remarks**
14	rs7308720	N551K (AAC>AAG )	Polymorphism
19		R767H (CGT>CAT)	Novel mutation Novel mutation
20		S885N (AGT>AAT)	Novel mutation
22	rs7966550	L953 ( TTA>CTA)	Polymorphism
30	rs7133914	R1398H (CGT>CAT)	Polymorphism
30	rs11175964	K1423 (AAG>AAA )	Polymorphism
31	ss48398558	R1441H (CGC>CAC)	Mutation*
31		R1483 ( CGA>AGA)	Novel variant
34	rs1427263	G1624 (GGC>GGA )	Polymorphism
34	rs33949390	R1628P (CGT>CCT)	Polymorphism
34	rs11176013	K1637 (AAA>AAG )	Polymorphism
34	rs11564148	S1647T ( TCA>ACA)	Polymorphism
37	rs10878371	G1819 (GGT>GGC )	Polymorphism
41		Y2018 (TAC>TAT )	Novel variant
42		N2047 (AAT>AAC )	Novel variant
43	rs10878405	E2108 (GAG>GAA )	Polymorphism
48	rs34778348	G2385R ( GGA>AGA)	Polymorphism
49	rs3761863	M2397T (ATG>ACG)	Polymorphism

* Reported [20–23]

### Case-control study of N551K, R1398H, R1628P, S1647T, G2385R and M2397T

A case–control study in a cohort of PD (n = 573) and ethnically matched controls (n = 503) was conducted to assess the association of the six amino acid-changing variants with risk of PD. The genotype distributions in PD and controls did not deviate significantly from Hardy–Weinberg equilibrium for any of the six variants examined (data not shown). The SNPSpD method was employed for correction of multiple SNP testing. SNPSpD output of six λs was shown in Table 2. As described by Cheverud [24], high correlation among variables leads to high λs. In this case, the first λ (2.37) was less than 6 (the number of variables in the correlation matrix), suggesting that not all variables are completely correlated. The magnitude of pair-wise LD was quantified by the metrics D’ and Δ^2^. The D’ and Δ^2^ coefficients of 551 and 1398 sites were 0.94 and 0.77, respectively, suggesting less historical recombination and more LD between 551 and 1398 sites. This was also true for 1647 and 2397 sites, with a D’ coefficient of 0.93 and a Δ^2^ coefficient of 0.59.

**Table 2:** Pairwise linkage disequilibrium measures for *LRRK2* SNPs.

		**D’**					
		**N551K**	**R1398H**	**R1628P**	**S1647T**	**G2385R**	**M2397T**
**Δ^2^**	N551K	***2.37***	0.94	1.00	0.96	0.88	0.72
	R1398H	0.77	***1.45***	1.00	0.93	0.98	0.77
	R1628P	0.00	0.00	***0.96***	0.96	0.04	0.87
	S1647T	0.05	0.05	0.04	***0.87***	0.89	0.93
	G2385R	0.00	0.00	0.00	0.05	***0.23***	0.94
	M2397T	0.05	0.07	0.02	0.59	0.04	***0.12***

Lewontin’s standardized disequilibrium coefficients (D’) are given above the diagonal and the squared pairwise correlations (Δ^2^) are given below the diagonal; the eigenvalues (λs) associated with the LD correlation matrix are given along the diagonal (*bold, italic*).

The genotype and allele distributions of the six variants for both patients and controls are outlined in Table 3. A statistically significant difference in G2385R A allele (4.6% vs. 2.1%, P = 0.0013) distribution between patients and controls was observed. When odds ratios of the at-risk genotype/allele were calculated, an increase in risk of developing PD was demonstrated for G2385R A allele (odds ratio: 2.27, 95% confidence interval: 1.38 - 3.88, P = 0.0017). The allele distribution of G2385R was further analyzed after being stratified by age. In the early onset PD (EOPD) group (AAO ≤ 50), a significant difference in G2385R A allele (5.1% vs. 0.8%, P = 0.0063) distribution between patients and controls was observed. EOPD patients with A allele have odds ratio 6.61 (95% confidence interval: 1.72 - 43.35, P = 0.0155) as compared with controls. In the late onset PD (LOPD) group (AAO > 50), a significant difference in G2385R A allele (4.5% vs. 2.5%, P = 0.0295) distribution between patients and controls was also observed. The LOPD patients with A allele has an odds ratio of 1.84 (95% confidence interval: 1.08 - 3.26, P = 0.0288) as compared with controls. The difference in A allele distribution between EOPD and LOPD groups were not significant (5.1% vs. 4.5%, P = 0.7329). The allele distribution of other variants did not show a significant difference between early and late onset PD patients groups as well as controls.

**Table 3:** Genotype and allele distribution and association analysis.

	**Frequency (%)**		***P*-value**			**Odds ratio(95% CI)**	***P*-value**
	**PD (n=573)**	**Controls (n=503)**					
Age (years)	62.1 ± 11.5	59.4 ± 12.9					
Sex (female)	44.7%	49.3%					
N551K					N551K		
CC, CG, GG	85.7, 13.6, 0.7	83.9, 15.7, 0.4	0.5118		CG+GG vs. CC	0.87 (0.62-1.21)	0.4134
G allele	7.5	8.3	0.5209		G allele	0.91 (0.67-1.25)	0.5770
R1398H					R1398H		
GG, GA, AA	84.3, 15.0, 0.7	80.9, 18.9, 0.2	0.1224		GA+AA vs. GG	0.79 (0.58-1.08)	0.1442
A allele	8.2	9.6	0.2413		A allele	0.84 (0.62-1.23)	0.2418
R1628P					R1628P		
GG, GC, CC	94.1, 5.9, 0.0	95.6, 4.4, 0.0	0.2504		GC vs. GG	1.38 (0.80-2.42)	0.2521
C allele	3.0	2.2	0.2568		C allele	1.37 (0.80-2.39)	0.2586
S1647T					S1647T		
TT, TA, AA	40.5, 46.4, 13.1	36.4, 49.7, 13.9	0.3851		TA+AA vs. TT	0.84 (0.66-1.08)	0.1675
A allele	36.3	38.8	0.2381		A allele	0.90 (0.76-1.07)	0.2381
G2385R					G2385R		
GG, GA, AA	90.8, 9.2, 0.0	95.8, 4.2, 0.0	0.0010		GA vs. GG	2.34 (1.41-4.02)	0.0014
A allele	4.6	2.1	0.0013		A allele	2.27 (1.38-3.88)	0.0017
M2397T					M2397T		
TT, TC, CC	29.3, 50.6, 20.1	25.8, 52.5, 21.7	0.4318		TC+CC vs. TT	0.84 (0.64-1.10)	0.2041
C allele	45.4	47.9	0.2391		C allele	0.90 (0.76-1.07)	0.2391

To examine if there is any haplotype of *LRRK2* 551, 1398, 1628, 1647, 2385 or 2397 site may associate with PD, pairwise haplotype analysis in the *LRRK2* gene was performed and the results (frequency ≥ 1%) are shown in Table 4. The 1647T-2397T haplotype was notably lower in PD patients than the controls (27.7% vs. 33.0%, *P* = 0.0244), with a trend toward decrease in risk of developing PD (odds ratio: 0.80, 95% confidence interval: 0.65 - 0.97, *P* = 0.0215). However, when G2385R was linked to 1647T-2397T (1647T-2385R-2397T haplotype), an increase in risk of developing PD (odds ratio: 2.15, 95% confidence interval: 1.27 - 3.78, *P* = 0.0058) was still observed, suggesting that 1647T-2397T haplotype cannot counteract the genetic effect of 2385R in PD. 

**Table 4:** Haplotype distributions of *LRRK2* polymorphisms in patients with Parkinson’s disease (PD) and controls and associations in PD risks.

**Haplotype** *		**PD / NC (%)**	***P*-value**	**Odds ratio (95% CI)**	***P*-value**
Wild type (N551-R1398-R1628-S1647-G2385-M2397)	000000	51.2 / 48.6	0.3923	1.00	
2397T	000001	3.7 / 2.6	0.1595	1.35 (0.82-2.25)	0.2479
1647T	000100	1.3 / 1.5	0.7210	0.83 (0.40-1.73)	0.6218
1647T-2397T	000101	27.7 / 33.0	0.0244	0.80 (0.65-0.97)	0.0215
1647T-2385R-2397T	000111	4.3 / 1.9	0.0019	2.15 (1.27-3.78)	0.0058
1628P-1647T-2397T	001101	2.8 / 2.0	0.2311	1.33 (0.76-2.40)	0.3243
1398H-2397T	010001	1.0 / 1.4	0.3538	0.65 (0.29-1.45)	0.2984
551K-1398H	110000	1.0 / 0.9	0.7209	1.11 (0.47-2.74)	0.8135
551K-1398H-2397T	110001	5.8 / 6.7	0.4504	0.83 (0.58-1.19)	0.3190

* Wild type = 0, variant = 1; examples: N551-R1398-R1628-S1647-G2385-M2397 nominated as 000000, 1647T-2397T nominated as 000101, 1647T-2385R-2397T nominated as 000111.

## Discussion

The present study consolidates the role of *LRRK2* G2385R as a risk factor of PD, and supports that S1647T-M2397T haplotype may lower the susceptibility of PD among Taiwanese population. We also identify one reported (R1441H) and two novel missense mutations (R767H and S885N) of *LRRK2*. Although the genome-wide association studies (GWAS) reported a strong association between *LRRK2* genetic variations and PD [25,26], the GWAS association signal has not been driven by identified missense variant as the G2385R, which may be due to this risk variant is ethnic specific.

The G2835R variant on the WD40 domain was first reported in a PD patient from Taiwan, with less than 1% frequency in Caucasian controls [20]. This variant is more common in Asia and is associated with an increased risk of PD in Japan, Singapore and Mainland China [27–30], in addition to Taiwan [7–9]. When over-expressed in human HEK cells, the G2835R variant was more toxic and associated with a higher rate of apoptosis under condition of oxidative stress [27]. Acting differently from the common LRRK2 kinase-activating G2019S mutation [31], the G2385R mutant causes a partial loss of the kinase function of LRRK2 [32]. In M17 neuroblastoma cell line, G2019S mutation decreased the average length of neurites and G2019S/G2385R double mutants counteract the neurite shortening effect of G2019S, suggesting that the impact of G2385R is strong enough to overcome the kinase-activating effect of the G2019S [32]. Since both loss and gain of kinase function variants are pathogenic, it is likely that the kinase activity of LRRK2 can be tolerated over only a narrow range. It is also possible that the G2385R mutation leads to pathogenic effects via other mechanism, which raises another therapeutic aspect for PD.

The protective *LRRK2* variants and haplotypes have been reported in PD patients. For example, R1398H and N551K reduce the risk of PD in Han-Chinese population [33]. Individuals carrying haplotype 551K-1398H-1423K have a significant reduction of PD risk in the white, Asian, and Arab-Berber populations [34]. Herein we identified a new *LRRK2* haplotype 1647T-2397T related to the reduced risk for PD, although results seen in single variant disease-association analysis does not find risk alterations in these two polymorphisms. S1647T is located at the highly evolution-conserved COR domain, which is thought to be a regulator of ROC GTPase activity [35]. In a Taiwanese study, S1647T is associated with increased PD risk, after considering the interaction effects with pesticide exposure [36]. These results contrast with the effect of 1647T-2397T to reduce PD risk, suggesting that other, yet unknown, molecular mechanisms are involved. Located on WD40 domain, M2397T is a risk-associated polymorphism in inflammatory bowel disease [37]. This variant decreases the amount of LRRK2 by altering the protein stability when expressed in HEK-293 cells [38]. This mechanism may contribute to its protective role in PD. As the risk of developing PD with 1647T-2385R-2397T haplotype is similar to that with 2385R allele alone, the protective effect of 1647T-2397T haplotype may be absent in the population carrying G2385R risk variant. Alternatively, the protect effect of 1647T-2397T may be attributable to the absence of G2385R variant. A larger cohort study will be needed to delineate the genetic effect of 1647T-2397T haplotype on PD risk reduction.

Two novel (R767H and S885N) and one reported (R1441H) missense mutations were identified in this population study. R767H is located in the ANK domain [6], which may play a role in protein folding [39]. Although the substitution of arginine with histidine would not dramatically affect the protein polarity, the newly added guanidine group may affect the protein stability by modifying the folding structure of LRRK2. S885N mutation substitutes serine with asparagine at the hinge between the ANK and LRR domains. The molecular mechanism of this mutation remains elusive. R1441H lies within the ROC GTPase domain, and more recently identified mutations affecting the same amino acid (R1441C, R1441G) have been described in affected PD patients [4,20]. R1441C mutation has been shown to increase LRRK2 kinase activity [31]. Both R1441C and R1441G mutations affect the GTPase activity of LRRK2 [40]. Lymphoblastoid cell lines carrying R1441H mutation showed increased apoptosis following exposure to proteasome inhibitor [41]. Thus, these mutations act dominantly and most likely cause enzymatic or structural gain-of-function that leads to neuronal toxicity.

Although our results are significant, there are limitations in this study. The role of gene-environmental interaction has not been evaluated. The sample size in our study may not be able to identify an association when the genetic effect of the allele is weak. This may explain the lack of protective effects of R1398H and N551K and increased risk of R1628P seen in a Chinese multicenter study [33]. Additionally, there is insufficient segregation to prove the pathogenicity of the two novel mutations (R767H and S885N). Nevertheless, our population study provides more information about the genetic variant of *LRRK2* in Taiwanese PD patients, and discovers two novel *LRRK2* mutations. Further study is needed to identify the functional implications of these genetic variants, which may shed light on developing new therapeutic strategies for PD. 

## Materials and Methods

### Ethics statement

This study was performed according to a protocol approved by the Institutional Review Board of Chang Gung Memorial Hospital (ethical license No: 97-2476A3), and all examinations were performed after obtaining written informed consents.

### Patient population

A total of 573 unrelated Taiwanese PD subjects (44.7% females) were recruited from the neurology clinics of Chang Gung Memorial Hospital (CGMH). All patients were diagnosed with probable idiopathic PD according to the published criteria [42] by two neurologists specialized in movement disorders (Y.-R. Wu and C.-M. Chen). Subjects with a prior history of multiple cerebrovascular events or other causes of parkinsonian symptoms (e.g. brain injury or tumor, encephalitis, antipsychotic medication) were excluded. The mean age at onset of PD was 62.1±11.5 years, ranging between 19 and 93 years. A group of 503 normal controls without neurodegenerative diseases were recruited from the same ethnic community. Control subjects (49.3% females) had mean age at examination of 59.4±12.9 years, ranging between 20 and 90 years. 

### Genetic analysis

Genomic DNA was extracted from peripheral blood lymphocytes using standard protocols. For PD patients with onset ≤ 50 (n = 70), RNA was extracted using PAXgene Blood RNA Kit (PreAnalytiX). The RNA was DNase (Stratagene) treated, quantified, and reverse-transcribed to cDNA using High Capacity cDNA Reverse Transcription Kit (Applied Biosystems). Using overlapping primers, *LRRK2* cDNA encompassing ANK, LRR, ROC, COR, MAPKKK and WD40 domains was polymerase chain reaction (PCR) amplified (Table 5), gel purified and sequenced directly using the ABI PRISM 3130 Genetic Analyzer (Applied Biosystems). The reported R1441H (ss48398558) and the novel R767H and S885N mutations were verified by genomic DNA PCR (Table 5) and sequencing. For population screening, the R767H and R1441H were examined using the *Bsp*HI (gain of site) and *Bst*UI (loss of site) restriction enzymes, respectively; amplification refractory mutation system (ARMS) PCR was designed for S885N population screening (Table 5). For case–control studies, the N551K (rs7308720), R1398H (rs7133914), R1628P (rs33949390), S1647T (rs11564148), G2385R (rs34778348) and M2397T (rs3761863) SNPs were determined using the *Ear*I (gain of site), *Bsp*HI (gain of site), *FspB*I (gain of site), *Afl*III (loss of site), *Acc*I (gain of site) and *Taa*I (gain of site) restriction enzymes, respectively (Table 5). In addition, primers and probes for allele specific primer extension assay (Table 6) were designed for N551K, R1398H and M2397T SNPs determination. 

**Table 5:** Primers and conditions for PCR amplification of *LRRK2* cDNA and genomic DNA.

Test (amplified region)	Anneal (°C) / MgCl_2_ (mM)	Product / RFLP enzyme (fragment, bp)
cDNA sequencing (ANK, LRR and ROC domain) F: TTGACTTAGTAATATTCCATCAAATGTCTTCC R: TCTCACTAGTTGTAATAATCGTTTCCGGTC	56 / 2.0	2663
cDNA sequencing (COR and MAPKKK domain) F: GAGAAGCAACGCAAAGCCTGCATGAGTA R: CCCATCTTCGGTATTGATGACCAGGAGAGTAC	66 / 2.0	2322
cDNA sequencing (WD40 domain) F: ACGTAATTGTTGAATGCATGGTTGCTACAC R: TTCAGGGTATCCACATTCAAACATAGAGTTG	65 / 2.0	1896
N551K (AAC>AAG ) (exon 14) F: tcacaaactggtcctagcag R: CCCCACTGTCATCTTATGTC	48 / 2.0	*Ear*I: GAAGAG (186 /166, 20)
R767H (CGT>CAT) (exon 19) F: CCCAGGTATCTTACAGTGAG R: GCCAAGAAGGTTCAACT	48 / 2.0	*Bsp*HI: TCATGA (573 / 419, 154)
S885N (AGT>AAT) (exon 20) F1:CAGAAGCATAGCAATACG F2:TGACTCTTCTATGGACAA R:CGTTCCAGTCTAGTCAGA	46 / 2.0	540, 308
R1398H (CGT>CAT) (exon 30) F: TAGGTACTTTGATCGGTTGCTGAC R: GACTTCATTACTCGGAAAGTTTCCC	52 / 2.0	*Bsp*HI: TCATGA (509 / 299, 210)
R1441H (CGC>CAC) (exon 31) F: GTGGCAGTCATATTTGCTTGAGTG R: ACCAGCCTACCATGTTACCTTGAA	56 / 2.0	*Bst*UI: CGCG (483, 232 / 715)
R1628P (CGT>CCT) (exon 34) F: TAGAGAAATTAGGTACTGTGTTGCACTT R: AAGAATAGATAGTGAATTTCCATGTAGC	56 / 1.5	*FspB*I: CTAG (157 / 83, 74)
S1647T (TCA>ACA) (exon 34) F: TAGGCCACATGGTTGCTAGAG R: CCTGCTTGGAACCAGCAAAT	54 / 1.5	*Afl*III: ACATGT (267, 82 / 349)
G2385R (GGA>AGA) (exon 48) F: TATAAGGTTGTATTACACGTAG R: TCTGAAAAGATGGTGCTGAGAAG	59 / 2.0	*Acc*I: GTAGAC (265 / 186, 79)
M2397T (ATG>ACG) (exon 49) F: CCAACAGGTCTCCTTGAT R: CCTAGCTGTGCTGTCATC	46 / 2.0	*Taa*I: ACNGT (754 / 553, 201)

**Table 6:** Primers and probes for allele specific primer extension (ASPE) assay of *LRRK2* N551K, R1398H and M2397T polymorphisms.

	Forward primer	Reverse primer	Probe
551 C/G	cagggaggatacagaatttcatc	ccccactgtcatcttatgtct	cctagcagctttgaa[C/G]
1398G/A	cggttgctgacaaatatgc	ctcgctgcgtcataaaatgg	[G/A]tgaggaattctatagtact
2397T/C	tggtggtggtgtcatgtttt	cctccagttcctatccaaagag	[T/C]ggtaaaagaaaacaagg

### Statistical analysis

The genotype frequency data and the expected genotypic frequency under random mating were computed and Chi-square tested for Hardy-Weinberg equilibrium using a standardized formula. The genotype and allele association analysis was carried out using the Chi-square test. The SNPSpD method [43] was used to generate an adjusted significance threshold for correction of multiple SNP testing (http://genepi.qimr.edu.au/general/daleN/SNPSpD/). The experiment-wide significance threshold of 0.0092 was required to keep the type I error rate at 5%. Measures of pairwise linkage disequilibrium (LD) between SNPs, including Lewontin’s standardized disequilibrium coefficients (D’), the squared pairwise correlations (Δ^2^), and eigenvalues (λs) were computed with the LDMAX software-part of the GOLD Command Line Tools package [44]. PHASE version 2.1 was used to infer the *LRRK2* gene haplotypes [45]. The *LRRK2* pairwise haplotype frequencies were computed and Chi-square tested for significance. Odds ratios with 95% confidence intervals (95% CI) were calculated to test association between genotype/allele/haplotype and disease.
